# Investigating Planning and Non-Targeted Exploration in PIAAC 2012: Validating Their Measures Based on Process Data and Investigating Their Relationships with Problem-Solving Competency

**DOI:** 10.3390/jintelligence11080156

**Published:** 2023-08-07

**Authors:** Maoxin Zhang, Björn Andersson, Samuel Greiff

**Affiliations:** 1 Centre for Educational Measurement, Faculty of Educational Sciences, University of Oslo, 0316 Oslo, Norway; bjorn.andersson@cemo.uio.no; 2 Department of Behavioral and Cognitive Sciences, University of Luxembourg, L-4366 Esch-sur-Alzette, Luxembourg; samuel.greiff@gmail.com

**Keywords:** log-file data, large-scale assessment, PIAAC, problem-solving, planning, non-targeted exploration

## Abstract

Problem-solving is a critical aspect of intelligence that has become increasingly important in modern society. Mapping out the determinants of success in problem-solving helps understand the underlying cognitive processes involved. This article focuses on two key cognitive processes in problem-solving: non-targeted exploration and planning. We generalize previously defined indicators of planning and non-targeted exploration across tasks in the 2012 Programme for the International Assessment of Adult Competencies and examine the internal construct validity of the indicators using confirmatory factor analysis. We also investigate the relationships between problem-solving competency, planning, and non-targeted exploration, along with the specific dependence between indicators from the same task. The results suggest that (a) the planning indicator across tasks provides evidence of internal construct validity; (b) the non-targeted exploration indicator provides weaker evidence of internal construct validity; (c) overall, non-targeted exploration is strongly related to problem-solving competency, whereas planning and problem-solving competencies are weakly negatively related; and (d) such relationships vary substantially across tasks, emphasizing the importance of accounting for the dependency of measures from the same task. Our findings deepen our understanding of problem-solving processes and can support the use of digital tools in educational practice and validate task design by comparing the task-specific relationships with the desired design.

## 1. Introduction

In modern societies, solving problems is a major task in our life (OECD 2014), involving multiple higher-order cognitive skills such as devising plans, testing hypotheses, remedying mistakes, and self-monitoring (Greiff et al. 2015). Thus, a high level of problem-solving competency lays a sound foundation for future learning and prepares students to handle novel challenges (Csapó and Funke 2017; OECD 2014). To make students better problem-solvers, it has been suggested to explicitly embed problem-solving skills into national curricula (Greiff et al. 2014) and use computer-based problem-solving simulations called “microworlds” where students can explore and discover underlying rules and regulations (Ridgway and McCusker 2003). Besides acquiring problem-solving competency in formal education, it is also important to develop such a skill over the entire lifetime and engage in lifelong learning (Greiff et al. 2013). For example, teachers might need to learn how to employ digital tools for long-distance education, and office workers might need to adapt to a different computer system. It has been documented that proficiency in applying information and communication technology (ICT) skills to solve problems has a positive influence on participation in the labor force (Chung and Elliott 2015). That is, the competency of problem-solving is both a key objective of educational programs (OECD 2014) and valued in the workplace.

Hence, many educational large-scale assessments for students and adults have focused on the domain of problem-solving. For example, the Programme for the International Student Assessment (PISA) evaluated 15-year-old students’ problem-solving in 2003, 2012, and 2015. Another example is the 2012 Programme for the International Assessment of Adult Competencies (PIAAC), which covers problem-solving in technology-rich environments when using ICT. Many of these assessments have been implemented on computers where the complete human–computer interactions are recorded in log files. Just as the task performance provides information on what respondents can achieve, the log files open a window into how respondents approach the task. Log files offer valuable information for researchers to understand respondents’ cognitive processes when solving problems, and this study intends to explore the log files of problem-solving tasks to infer the cognitive processes when solving problems. 

A better understanding of the problem-solving processes has potential implications for integrated assessments and learning experiences (Greiff et al. 2014). For example, the analysis results from log files can provide teachers with materials on the weaknesses and strengths of students in solving a problem, and further, teachers can tailor their instruction for students. In this study, we aim to improve the understanding of the cognitive problem-solving processes in the context of information processing. This can potentially benefit educational practices related to improving problem-solving skills. For example, the analysis of log files can inform teachers whether a student is engaged in solving a problem or applies an efficient strategy to approach the problem (Greiff et al. 2014) and whether additional instructional scaffolding is needed when a student is stuck. 

The data availability of international large-scale assessments has stimulated studies that explore the information from the log files. Both theory-based methods (e.g., Yuan et al. 2019) and data-driven methods based on machine learning or natural language processing (e.g., He and von Davier 2016) have been applied to extract information called process indicators from log files, and the relationships between these process indicators and task performance have then been inferred. However, the majority of research has focused on single tasks, and the generalizability of the conclusions remains unclear. In this study, we used process indicators to analyze multiple tasks involving two cognitive aspects of problem-solving: planning and non-targeted exploration. Specifically, we examine the internal construct validity of the measures of planning and non-targeted exploration using tasks from PIAAC 2012 and infer their relationships with problem-solving competency. Next, we review the literature on problem-solving, planning, and non-targeted exploration and describe the current study in more detail.

### 1.1. Problem-Solving

A problem is considered to have two attributes: (a) the difference between a given state and the desired goal state and (b) the social, cultural, or intellectual worth embedded in achieving the goal (Jonassen 2000). Problems can be categorized into different types according to their characteristics. Here, we introduce three problem categories based on dynamics, structuredness, and domain (Jonassen 2000). First, problems can be categorized as static or dynamic problems based on the dynamics of a problem situation. In static problems, all the information relevant to the problem is known at the outset (Berbeglia et al. 2007). In contrast, dynamic problems (also called complex problems) do not present all the necessary information at the outset; instead, problem-solvers must interact with the problem situation to collect relevant information (Stadler et al. 2019). Thus, exploring the problem situation plays a more important role in dynamic problems compared with static problems. In addition, according to the structuredness (i.e., the clarity of a problem), a problem can be mapped into a curriculum with two poles representing well-structured and ill-structured problems (Arlin 1989). Problems in textbooks tend to be well-structured problems with a clearly defined initial and goal state and operator rules, whereas problems such as designing a building are ill-structured problems. The tasks in PISA 2012 and PIAAC 2012 are relatively well-structured problems, and the optimal solutions are predefined. Moreover, based on the specific domain knowledge required to solve a problem, problems can be categorized as domain-specific and domain-general (Jonassen 2000). For example, physics and biology exams typically present domain-specific problems. In contrast, finding a quickest route between two places and figuring out why a lamp is not working are examples of domain-general problems in everyday contexts.

**Figure 1:**
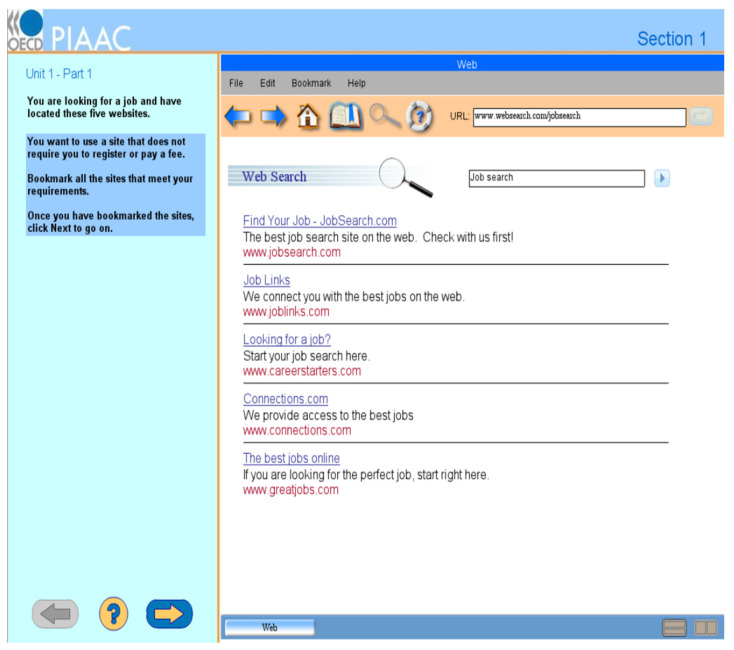
An example task released from the PIAAC PS-TRE domain. The figure was retrieved from https://piaac-logdata.tba-hosting.de/public/problemsolving/JobSearchPart1/pages/jsp1-home.html (accessed on 19 October 2021).

The cognitive process of transferring a given state into a goal state when the solution is not immediately accessible is called problem-solving (Mayer and Wittrock 2006). Mayer and Wittrock (2006) argued that problem-solving involves several component processes: representing, planning/monitoring, executing, and self-regulating. We take a problem-solving task released from the PIAAC 2012 (see Figure 1) as an illustrative example. The task requires participants to bookmark job-seeking websites that do not need registration or fees. When confronted with this problem, respondents must convert the given information into a mental representation, which includes the initial state (e.g., five website links in this example), goal state (e.g., bookmarked websites satisfying the requirements), and the possible intermediate states (Bruning et al. 2004). Such a process is called representing. Planning occurs when respondents devise a way to solve the problem (Mayer and Wittrock 2006), such as decomposing it by checking the links from the first to the last to see which require registration or a fee. Monitoring refers to the process of evaluating whether the solution is valid and effective (Mayer and Wittrock 2006). Implementing the planned operations is called executing (Mayer and Wittrock 2006). Self-regulating involves modifying and maintaining activities that allow respondents to move toward the goal (Schunk 2003). While these processes are all assumed to be active in problem-solving, the importance of each cognitive process differs across problems.

In a technology-rich society, problems often appear because new technology is introduced (OECD 2012). On the other hand, tools and technologies are widely applied to facilitate problem-solving. Capturing the intersection of problem-solving competency and the skills needed in ICT, the 2012 PIAAC specifically covers a domain called problem-solving in technology-rich environments (PS-TRE), where problem-solving competency is defined as the capacity of “using digital technology, communication tools and networks to acquire and evaluate information, communicate with others and perform practical tasks” (OECD 2012, p. 47). The 2012 PIAAC PS-TRE domain developed fourteen problems that are dynamic, relatively well-structured, and domain-general information problems. The problems are assumed to assess a single dimension—problem-solving competency (OECD 2012). In addition to problem-solving competency, PIAAC 2012 also emphasizes the cognitive dimensions of problem-solving. The PS-TRE domain shares similar cognitive problem-solving processes with Mayer and Wittrock (2006) but with a particular focus on acquiring and dealing with information in computer-based artifacts. 

To acquire the relevant information, it is necessary to interact with the problem environment and explore the features or potential resources that are closely related to the representing process. After collecting useful information, respondents may devise a plan (e.g., to break down the problem and set sub-goals for achieving the desired state). These two processes, exploration and planning, play vital roles in problem-solving and are thus the focus of this study. We next introduce the definitions and measures of planning and exploration (particularly non-targeted exploration) and their relationships with task performance.

### 1.2. Planning and Problem-Solving

Planning is defined as mental simulations of future operations and associated outcomes with the aim of achieving certain goals or guiding problem-solving (Mumford et al. 2001). An early conception of planning referred to certain predefined, fixed sequences of operations. More recently, however, researchers have argued that adaptable cognitive responses are at the core of planning (Mumford et al. 2001). In addition, it is assumed that planning consists of multiple and distinguishable processes (Hayes-Roth and Hayes-Roth 1979). For example, Mumford et al. (2001) proposed a planning process model: prior to developing an initial and general plan, environment analyses including the identification of resources and contingencies are necessary. Then, an initial plan needs to be elaborated into a more detailed plan, which requires searching information about potentially useful operations and resources needed to execute these operations (Xiao et al. 1997). Based on the forecasting of outcomes from these operations, one may refine the plan and then execute it.

Planning is a generative activity that is hard to observe directly. Early qualitative studies applied think-aloud protocols and content analyses to investigate planning (e.g., Xiao et al. 1997). Recently, quantitative measures have been used to facilitate research on planning, such as evidence from functional neuroimaging (Unterrainer and Owen 2006) and time-related measures (Albert and Steinberg 2011; Eichmann et al. 2019; Unterrainer et al. 2003). In this study, we consider the process measure of response times as an indicator of planning. Because planning is resource-intensive (Mumford et al. 2001), the time spent making a plan should be much longer than the time spent actually executing the plan. The time-related measures capture the quantity of planning. If a respondent rushes into a problem and randomly tries different operations until a correct solution is found (i.e., a trial-and-error strategy), the value of the time-related measures would be relatively small, indicating a small quantity of planning.

In the context of problem-solving, the time-related measures of planning differ between static problems and complex problems. A commonly used measure of planning in static problems, such as the Tower of London, is the first-move latency (Albert and Steinberg 2011; Unterrainer et al. 2003). This measure, also known as preplanning time, is defined as the time interval between the beginning of the problem and the first action a respondent takes. However, in complex problems, respondents need to explore the simulated environment to generate information before they are able to make a plan that takes into account all relevant aspects of the problem situation at hand. In line with this thinking, Eichmann et al. (2019) expanded the measure of planning in complex problems from the first-move latency to the longest duration between moves. Namely, the authors argued that planning can appear at any time during the course of complex problem-solving. They also acknowledged that the longest duration cannot cover the entire planning process but that the main planning activity is captured by this indicator. Research on planning in complex problems is quite limited, and Eichmann et al.’s (2019) work seems to be the first on this topic, thus, yielding important implications for the current study.

Planning is of interest not only because it is a cognitive process in problem-solving but also because it influences task success or task performance (Albert and Steinberg 2011; Eichmann et al. 2019). Theoretically, planning provides a mental model of the problem by identifying critical issues and relevant strategies and promotes optimized and effective solutions by organizing the chunks of operations (Mumford et al. 2001). However, previous empirical research showed diverse results regarding the relationship between task success and planning due to different types of problems and different indicators of planning. For instance, and as mentioned above, Albert and Steinberg (2011) found a positive relationship between first-move latency and task success in static problems, whereas Eichmann et al. (2019) did not find such an effect for the longest duration indicator in dynamic problems. Additionally, Eichmann et al. (2019) derived two other indicators of planning to describe the time taken before the longest duration appears (the delay indicator) and the variability in time intervals between two successive operations (the variance indicator). They found that planning in the early stages benefited task performance (i.e., a negative relationship between the delay indicator and task scores) and that a longer duration indicator in a later stage or continued planning activities could compensate for a lack of early planning. Their models implicitly indicate that each indicator from different tasks implies similar meanings (Assumption I) and that the relationships between the planning indicators and task success are consistent across tasks (Assumption II). However, we argue that these assumptions (i.e., Assumptions I and II) require explicit examination. In addition, although the random effects in their models captured the variances at the task level, the specific relationships between the indicators and task performance at the task level remained unaccounted for.

### 1.3. Non-Targeted Exploration and Problem-Solving

To better understand the nature of the problem, test-takers need to explore the problem environment (e.g., navigate through different computer interfaces or pages) to uncover new information. Exploration refers to behaviors that investigate and seek information that is beyond the instructions of the task (Dormann and Frese 1994). Some exploratory behaviors are goal-oriented (goal-directed behaviors), leading to achieving a desired goal state. On the other hand, some exploratory behaviors can be irrelevant to solving the problem (non-targeted behaviors), such as clicking on some buttons on the interface to check their functions and exploring some pages that do not contain useful information for the problem (Eichmann et al. 2020a, 2020b). Note that both goal-directed and non-targeted behaviors help test-takers understand the problem but in different ways. Goal-directed behaviors capture the relevant points and convey similar information as task success because the problem cannot be successfully solved without these goal-directed behaviors, whereas non-targeted behaviors provide additional information compared to task success.

One research field related to non-targeted exploration is error management, where errors are defined as unintended deviations from goals (Frese et al. 1991). It is found that compared to participants who received step-by-step guidance on programming (i.e., error avoidance or goal-directed exploration), participants who were encouraged to explore the system, make mistakes, and learn from them (i.e., non-targeted exploration) during the training stage performed better during the testing stage (Frese and Keith 2015). One explanation is that non-targeted exploration plays a role in representing the problem (Eichmann et al. 2020b; Kapur 2008). Test-takers who were encouraged to explore the environment, in spite of making more errors, gained a better understanding of the problem setting, the potential features, and resources of the interfaces. In addition, participants who received more training on exploratory error management showed a higher level of metacognitive activity such as hypothesis-testing and monitoring (Keith and Frese 2005).

In computer-based problems, exploration is operationalized as human–computer interactions that refer to all the operations that respondents conduct in the computer system and are recorded in log files, such as mouse clicks and keyboard input. For each item, test developers and content experts have predefined one or more optimal solutions consisting of a minimum number of operations that can successfully solve the problem and thus represent the most efficient strategies (He et al. 2021). We can broadly categorize individual operations into goal-directed or non-targeted operations, depending on whether the operation is required to solve the problem or not (Eichmann et al. 2020a, 2020b). Goal-directed operations refer to operations that must be performed to solve the problem, which are operationalized as the operations that occur in any of the optimal solutions. In contrast, non-targeted operations are operations that are unnecessary to solve the problem, which are operationalized as the operations that do not occur in any optimal solutions. For example, in the task of Figure 1, clicking on and bookmarking the websites that satisfy the task requirements are goal-directed operations. However, clicking on the Help button in the menu is non-targeted because it is not included in the optimal solution. 

Although non-targeted operations do not directly contribute to successful task completion (i.e., not occurring in any optimal solutions) and can appear erroneous, they have been found to benefit task performance (Dormann and Frese 1994), learning (Frese and Keith 2015), transfer performance (Bell and Kozlowski 2008), and meta-cognition (Bell and Kozlowski 2008). Eichmann et al. (2020a) also found that the number of non-targeted explorations is positively related to problem-solving competency, and the effects are consistent across 42 countries using the PISA 2012 problem-solving domain. The authors argued that non-targeted explorations facilitate goal-directed behaviors. Consider the Help button as an example. Although the Help button is not considered as a necessary operation to solve the problem, it provides test-takers with information about the functions of the menu, such as the function of the bookmark button, which can help test-takers better understand the potential resources in the computer system. When test-takers find the websites that meet the task requirements, they would know how to bookmark the websites.

A further aspect of defining an operation is whether it is performed for the first time or repeated. Implementing an operation for the first time is associated with information generation, whereas performing the same operation again indicates information integration (Wüstenberg et al. 2012). As a result, Eichmann et al. (2020b) distinguished between initial and repeated operations. Once a respondent performed a specific operation, such as clicking on the Help button in the task in Figure 1, the individual was assumed to gain information related to the Help button. If the respondent performed the same operation again, there would be little new information added to the problem space. Since exploration greatly concerns generating new information (Dormann and Frese 1994), we propose the number of initial non-targeted operations as a measure of the latent variable: non-targeted exploration. This differentiates our study from Eichmann et al. (2020b), who focused on both initial and repeated non-targeted operations. 

### 1.4. The Current Study

Previous studies by Eichmann and coauthors have deepened the understanding of planning and non-targeted exploration based on the PISA 2012 tasks (Eichmann et al. 2019, 2020a). However, the extent to which we can apply their definitions of planning and non-targeted exploration to the PIAAC 2012 information problems and the extent to which the indicators measure the same constructs require further research. If there is insufficient evidence of internal construct validity, it would be problematic to apply this measure to different items or different samples. Therefore, validating the internal construct of planning and non-targeted exploration across items is a crucial component of this study. We concurrently utilize information from multiple tasks and validate the approach of Eichmann and coauthors by looking at a more diverse set of tasks (i.e., PS-TRE) with a different population, namely, adults.

Furthermore, most studies analyzing process data of problem-solving tasks have only used log data from a single item (e.g., Ulitzsch et al. 2021; Chen et al. 2019), meaning the generalizability of the findings to other tasks is lacking. For example, it is an open question whether or not respondents apply similar strategies (e.g., trial-and-error) across tasks. Similarly, are the relationships between planning and problem-solving competency stable across tasks or are the relationships task-dependent? If the relationships are generalizable, then researchers and practitioners can use the findings across similar tasks. In this study, we examine the general and task-specific relationships between planning, non-targeted exploration, and problem-solving competency.

Our first set of research questions concerns the internal construct validity of the indicators for planning, non-targeted exploration, and problem-solving competency. If we find evidence that the same operationalization (see detailed definitions in Section 2.3) of the indicators is applicable across different items within different contextual settings, this implies that the indicators measure the same construct, thus providing support for internal construct validity for the indicators. Specific to the current study, we examine the construct validity of planning (*Q1a*), non-targeted exploration (*Q1b*), and problem-solving competency (*Q1c*) using a set of tasks from the PIAAC 2012 PS-TRE domain. For each item, we extract the indicators for planning, non-targeted exploration, and problem-solving competency along the same rationale. To examine evidence of construct validity, we applied confirmatory factor analysis (CFA; Jöreskog 1969) to each type of indicator. In CFA models, multivariate data are analyzed with the hypothesis that a latent variable underlies the observed variables (Bartholomew et al. 2011, p. 2). For example, the item response score is considered to be the observed indicator of the latent variable problem-solving competency. If the variations of the indicators across items can be adequately attributed to a latent variable, we can claim that the internal construct validity is established (AERA 2014). 

The second set of questions that we are interested in points to the problem-solving competency’s relationship with planning (*Q2a*) and non-targeted exploration (*Q2b*). Although previous studies have investigated such questions (e.g., Albert and Steinberg 2011; Unterrainer et al. 2003), only limited studies have examined the findings in dynamic problems (Eichmann et al. 2019, 2020b). Given that dynamic problems are becoming more popular in educational assessments and that the planning and exploration processes might differ between static and dynamic problems, examining their relationships with problem-solving competency is relevant and needed. In the research of Eichmann et al. (2019), the overall relationship between planning and task performance across tasks was examined, whereas if such a relationship might differ between tasks was uncounted for. Tasks differ in complexity, the interface, and the amount of information (OECD 2013), implying that the importance of planning and non-targeted exploration varies among the tasks. Hence, besides the overall relationships between the latent variables (i.e., planning, non-targeted exploration, and problem-solving competency), we also consider their task-specific relationships by adding residual correlations of observed indicators for planning, non-targeted exploration, and problem-solving competency from the same task. The variance of the errors can be attributed to individual differences among participants, task characteristics, and measurement error. The residual correlations that we added account for the additional dependence between indicators based on the same task, beyond the dependence induced by the correlations between the main factors of planning, non-targeted exploration, and problem-solving competency. Hence, by answering *Q2a* and *Q2b* from the levels of both latent variables and observed variables, we can gain a more fine-grained understanding of the research questions than Eichmann et al. (2019, 2020a). For *Q2a*, we hypothesized that the overall relationship between planning and problem-solving competency is negligible but that the relationship at the observed variable levels can be task-dependent, based on the results from Eichmann et al. (2019) and the diversity of tasks. For *Q2b*, because non-targeted exploration helps represent the problem and acquire information from available resources, we hypothesized a positive relationship between problem-solving competency and non-targeted exploration. Similarly, task-dependent relationships are also expected for *Q2b* because tasks differ in the extent to which respondents are allowed to interact with the interfaces. To achieve answers for *Q2a* and *Q2b*, we included all three indicators in a single model and considered the dependencies among the latent variables (i.e., the overall relationships) and the pairwise residual correlations of the three indicators from the same task (i.e., task-dependent relationships).

## 2. Materials and Methods

### 2.1. Participants and Tasks

This study uses the performance data and associated log files from the 2012 PIAAC assessment. PIAAC is a program for assessing and analyzing adult skills and competencies that are essential to personal and societal success (OECD 2013). The stimuli materials were developed based on everyday life activities, and the target population was noninstitutionalized residents between 16 and 65 years of age in the country regardless of citizenship or language (OECD 2013). The PIAAC assessment was implemented by 25 countries (OECD 2012). All participating countries produced their sample design under the guidance of the PIAAC Technical Standards and Guidelines. In general, probability-based sampling methods were adopted to select an unbiased, randomized, and representative sample of the target population (OECD 2013). Countries developed their own sampling frames according to national situations. For example, Singapore had a full list of residents in the population registry that was used as a qualified sampling frame, and the sample was randomly selected based on the population registry. However, many countries like the United States adopted a multi-stage sampling method since such population registries did not exist there. In short, geographic domains such as provinces or states and dwelling units were randomly selected in primary stages, and persons in the domains had an equal probability to be sampled at the last stage of selection. After obtaining a sample, checks were conducted to ensure that the sample met the sampling plan. For example, the noncoverage rate of the target population was computed to indicate the portion of the target population not covered by the sample frames. In the United States, people who live in large, gated communities are not covered, and the noncoverage rate is 0.1%, which is the lowest in all participating countries (OECD 2013). For a more detailed description of the sampling design, readers are directed to the PIAAC technical report (OECD 2013). To avoid cultural heterogeneity and render the analyses of the vast log-file data manageable, we used only data from the United States. We chose the sample from the United States because of the low noncoverage rate, high response rates, and the large proportion of participants in the PS-TRE domain.

The 2012 PIAAC PS-TRE domain covers dynamic information problems that include one or more digital scenarios (e.g., email, web, word processor, and spreadsheet). Each PS-TRE task includes two panels (see Figure 1): The left panel shows the instructions that describe the scenario and the goal state (i.e., bookmarked websites fulfilling some requirements), and the right one represents the initial problem environment that corresponds to the given state. Respondents may need to first explore the system by, for example, clicking on the menu or a link to get to know the problem environment and then spend a relatively long time devising a plan to solve the problem. There are two booklets in PS-TRE, and each consists of seven fixed-order tasks. Based on the assessment design, test-takers randomly received zero, one booklet, or two booklets. We used the second booklet (PS-TRE2). Only participants with sufficient ICT skills in the background questionnaire had access to the PS-TRE tasks. Sufficient ICT skills include knowing how to manipulate the mouse and keyboard, understanding concepts like files and folders, and having experience with basic computer operations like save, open, and close files (OECD 2013).

### 2.2. Data Preparation

The log files of the 2012 PIAAC domains can be downloaded from the GESIS Data Catalogue (OECD 2017). There were 1355 American participants in PS-TRE2, but 30 of them directly skipped all seven tasks and were excluded from the current analysis. The raw log files were preprocessed via the PIAAC LogDataAnalyzer (LDA) tool. The reformatted log data consisted of the following variables: respondent ID, item information, event_name, event_type (e.g., START, TOOLBAR, TEXTLINK), timestamp in milliseconds, and event_description, which describes the specific event (e.g., “id=toolbar_back_btn” means clicking on the back button in the toolbar). We recoded the data by filtering the system logs and aggregating the keyboard input and clicks in pop-up windows. A detailed explanation of this procedure is provided in Appendix A.

### 2.3. Measures

For each student on each item, we extracted three indicators: task scores, longest duration, and the number of initial non-targeted operations, from performance data and the log files. In this subsection, we describe the three measures in detail.

*Problem-solving competency.* The indicators for problem-solving competency were response scores that can be extracted from the OECD website. In PS-TRE2, three items were scored dichotomously, and four were scored polychotomously by PIAAC. If a participant spent less than five seconds on a task, the response was scored as missing (OECD 2012). In the current data set, only five response scores were denoted as missing values by PIAAC. We directly used their scoring as the measures for the construct problem-solving competency.

*Planning.* We used the time intervals between consecutive events from log files to compute the longest duration, excluding the time interval for the last two events. The last two events are always NEXT_INQUIRY (request the next task) and END (end the task) based on the task design, and the intervals for the last two operations indicate reflection on the executed actions rather than planning. A simulated operation sequence and associated time intervals for the job-seeking task are presented in Table 1. Excluding the time intervals for the last two operations, we identified the longest one—10 s—as the longest duration indicator. For those who directly skipped a task, the longest duration was coded as missing. In a previous study, Eichmann et al. (2019) specified three indicators of planning: the longest duration, the variance indicator, and the delay indicator. However, we found the Pearson correlations between the indicators were around 0.80 for the PS-TRE tasks, and the longest duration typically occurred just after the task began, which meant that the delay indicator was often identical to the duration indicator. That is, the three aspects of planning from Eichmann et al. (2019) largely overlapped in our data, and we therefore used only a single planning indicator per item for the construct planning in this study.

*Non-targeted exploration*. To define the non-targeted exploration indicators, we first identified the unique operations for each task based on the log files of the participants. There were on average 200 unique operations (range = [57, 446]) in each of the PS-TRE2 tasks. Operations that occurred in any of the optimal solutions were considered goal-directed operations and the others non-targeted operations. Thereafter, we defined the indicator of non-targeted exploration as the number of initial non-targeted operations for each item. For the Figure 1 example, we supposed that the correct solution was {START, textlink_page5, toolbar_bookmark_btn, bookmark_add_page5, NEXT_INQUIRY, END}. By subsequently checking whether a given operation in Table 1 was included in the optimal solution, we identified goal-directed or non-targeted operations. The number of initial non-targeted operations, which was three in this example, served as the indicator of non-targeted exploration. For those who directly skipped a task, the indicator was coded as missing.

*Data transformation.* Latent variable modeling like factor analysis for continuous data (Jöreskog 1969) normally has the assumption of multivariate normality, but both process indicators (i.e., longest duration and the number of initial non-targeted operations) deviated from normal distributions according to large skewness and kurtosis (see Appendix B), requiring data transformation. One approach is the Box–Cox transformation (Box and Cox 1964). However, such one-to-one transformations do not work well when the data have many identical values (Peterson and Cavanaugh 2019). In addition, there are some extreme outliers in the longest duration and the number of initial non-targeted operations. Instead of transforming the indicators into normally distributed variables, we used quantiles to recode the process indicators into equal-sized categorical variables, which can reduce the impact of the outliers. Specifically, if the raw value was zero, we kept the value as it was; for the remaining values, we recoded the values as 1, 2, 3, and 4 with the 25%, 50%, and 75% quantiles as the cutoff values. Higher categories indicate that more initial non-targeted operations were applied, or a respondent spent more time planning than other respondents. In the following analysis, we treat the three types of indicators (response scores, longest duration, and the number of initial non-targeted operations) as ordered categorical data.

### 2.4. Analysis Procedures

In this study, we apply latent variable models to analyze the process indicators and task performance. Latent variable models are widely used in social sciences when researchers intend to measure a conceptual construct (Bartholomew et al. 2011) such as problem-solving competency. However, since it is difficult to measure the construct directly, researchers instead develop instruments based on theory to infer the construct indirectly. In PIAAC 2012, a battery of items was developed to measure problem-solving competency, and respondents’ responses to the test are collected and considered as observed indicators of the unobserved construct (i.e., problem-solving competency). In analyzing the observed responses, the researchers extract what is common in the indicators. The latent variable that explains the common variability of the observed indicators is then interpreted as the problem-solving competency afterward. A similar approach is used to measure the latent variables of planning and non-targeted exploration, where the longest duration and the number of initial non-targeted operations from multiple items are used as observed indicators, respectively.

To answer the research questions related to the internal construct validity (i.e., *Q1a*/*Q1b*/*Q1c*), we applied confirmatory factor analysis (CFA; Jöreskog 1969) to each type of indicator. CFA is widely used to examine the latent construct by specifying the relationships between the observed indicators and latent variables on the basis of specific hypotheses (Brown 2015). We hypothesized that latent planning would underlie the longest duration (Model 1a), latent non-targeted exploration would underlie the number of initial non-targeted operations (Model 1b), and latent problem-solving competency would underlie the observed task scores (Model 1c). That is, the latent variables govern the associated observed indicators and thus explain the common variability of the indicators. To test these hypotheses, we examine if the hypothetical models fit well with the real data by checking the goodness-of-fit of the models and factor loadings that inform on the relationship between the observed indicators and the latent variable.

**Figure 2:**
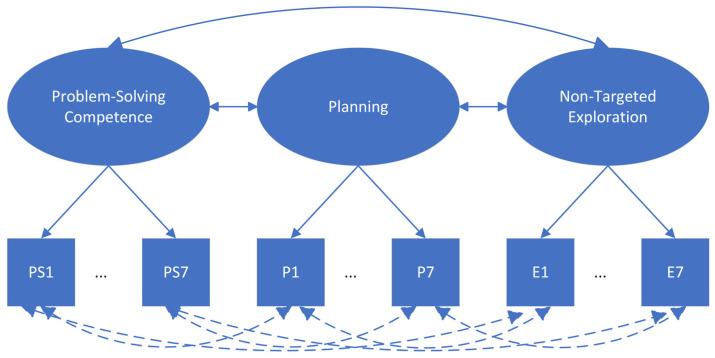
An illustration of Model 2. Note. P = planning indicator (i.e., longest duration); PS = task scores; E = non-targeted exploration indicator (i.e., the number of initial non-targeted operations). The numbers 1 to 7 indicate the position of the task in the booklet. Ellipses = latent variables; Rectangles = observed variables. The solid lines with double arrows indicate the covariance between the latent variables. The dashed lines with double arrows indicate the residual correlations between observed indicators.

Regarding *Q2a* and *Q2b*, we inferred the relationships between planning, non-targeted exploration, and problem-solving competency via multidimensional latent variable analysis (Model 2; see Figure 2). That is, we placed the three latent variables together with their correlations at the latent variable level (see the solid arrows between the latent variables in Figure 2) and pairwise residual correlations at the observed variable level (see the dashed arrows between the observed indicators in Figure 2). The covariances between problem-solving competency and planning and between problem-solving competency and non-targeted exploration address *Q2a* and *Q2b* at the latent variable level, respectively. A positive covariance would imply that, generally speaking, planning more or conducting more non-targeted operations is positively related to problem-solving competency. Given the diversity of tasks (e.g., interfaces and complexity), the answers to *Q2a* and *Q2b* might differ between tasks. Hence, we added pairwise residual correlations between the three indicators if they were derived from the same task. For example, for Task 1, we included the residual correlations between P1, E1, and PS1. These residual correlations help explain task-specific relationships among the indicators not captured by the covariances between the latent variables. For example, it could be possible that the overall relationship between non-targeted exploration and problem-solving competency is positive, but for certain tasks exploring more impairs task performance, namely negative task-specific relationships. The specified model is similar to De Boeck and Scalise’s (2019) model, which used time-on-task, the number of actions, and responses as indicators of latent speed, latent action, and latent performance, respectively, in the domain of PISA 2015 collaborative problem-solving. They also considered specific hypotheses about relationships between the residuals of the indicators that were based on the same tasks.

To estimate the models, we used the lavaan package (Rosseel 2012) in R 4.1.0 (R Core Team 2013) with the diagonally weighted least squares (DWLS) estimator and treated the observed data as ordered categorical variables. Missing values were handled by pairwise deletion. By convention, the means and variances of the latent variables were constrained as zeros and ones for the purpose of model identification, respectively. We evaluated the model fit with a robust chi-square test of fit and used the criteria the root mean square error of approximation (*RMSEA*) and the standardized root mean square residual (*SRMR*). *RMSEA* assesses how far a specified model is away from an ideal model, and *SRMR* evaluates the difference between the residuals of the model-implied covariance matrix and the observed covariance matrix. Hence, the lower *RMSEA* and *SRMR* are, the better the model fit with the data. The cutoff values are 0.06 and 0.08 for *RMSEA* and *SRMR*, respectively (Hu and Bentler 1998).

## 3. Results

We begin this section with a description of the sample characteristics. Among the 1325 participants, the average age was 39 years old (SD = 14), and 53% were female. Around 9%, 40%, and 51% of the participants’ highest level of schooling was less than high school, high school, or above high school, respectively. For the employment status, 66% of the participants were employed or self-employed, 3% retired, 8% not working and looking for work, 11% students, 6% doing unpaid household work, and 6% other jobs. PIAAC categorized respondents’ performance on the PS-TRE domain in four levels: less than level 1 (19% in the US dataset), level 1 (42% in the US dataset), level 2 (36% in the US dataset), and level 3 (3% in the US dataset). Higher levels indicate better proficiency.

**Figure 3:**
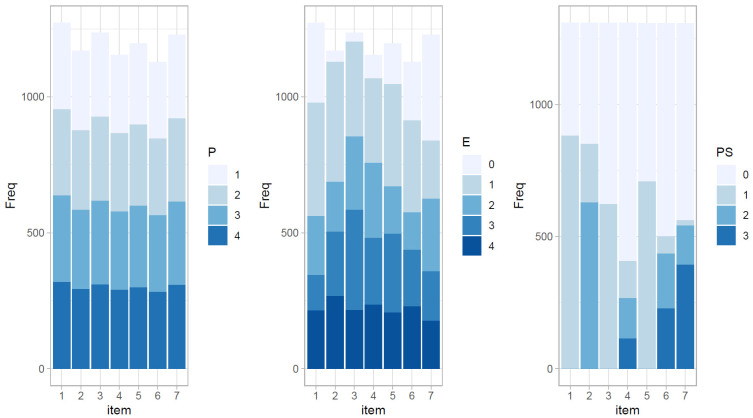
The frequency plot of planning (P), non-targeted exploration (E), and problem-solving competency (PS) indicators. The longest duration could not be zero, so the categories of the planning indicator consisted of only four values.

With respect to the responses on the PS-TRE tasks, some omission behaviors were observed for the tasks. There were on average 127 participants (range = [53, 197]) who did not interact with single tasks and requested the next task directly. Figure 3 plots the frequency of the derived indicators after the recoding procedure. The distributions of the planning indicator were almost evenly distributed across the four categories. However, the distributions of the other indicators were somewhat diverse depending on the items. For example, only a small proportion (2.4%) of participants did not try any non-targeted operations in Task 3, but more than one fourth (29%) did not explore Task 7.

Next, we present the results relevant to *Q1a*, *Q1b*, and *Q1c* based on the single-factor CFA models for planning (Model 1a), non-targeted exploration (Model 1b), and problem-solving competency (Model 1c). Table 2 presents the model fit indices and the standardized results for factor models. For the planning measurement model, although the robust chi-square test was significant (*p* = .013), the model fit indices (*RMSEA* = 0.021 (*se* = 0.006); *SRMR* = 0.042 (*se* = 0.003)) were lower than the cutoff values 0.06 and 0.08 (Hu and Bentler 1998), thus indicating good approximate model fit. All the factor loadings in Model 1a were significant, ranging from 0.491 to 0.691. The higher factor loading indicates a stronger relationship between the indicator and the latent variable, and thus the latent variable can account for more of the variability of the indicator. The results for the model fit and factor loadings provided evidence of validity for the construct planning. This conclusion also applied to the measurement model (Model 1c) for problem-solving competency (*RMSEA* < 0.001 (*se* = 0.003); *SRMR* = 0.020 (*se* = 0.003); nonsignificant chi-square test, *p* = .901). The factor loadings ranged from 0.636 to 0.813. For the non-targeted exploration measurement model (Model 1b), the model fit indices (*RMSEA* = 0.014 (*se* = 0.007); *SRMR* = 0.044 (*se* = 0.004)) were satisfactory, and the robust chi-square test was nonsignificant (*p* = .134). However, the factor loadings varied a lot (see Table 2). Tasks 3 and 4 had the highest factor loadings, whereas the last two tasks had the lowest with values less than 0.2. That is, although the non-targeted exploration indicators in PS-TRE2 generally measure the same construct, the impact of the latent non-targeted exploration on the observed indicators differed across tasks.

Subsequently, we present the results of Model 2. If we ignored the residual correlations of the indicators (i.e., the task-dependent effect), the model fit indices exceeded the cutoff values (*RMSEA* = 0.071 > 0.06, *se* = 0.002; *SRMR* = 0.096 > 0.08, *se* = 0.002). This suggests that only considering the overall relationships between the latent variables and excluding the task-dependent relationships did not fit well with the data. In Model 2, the residual correlations were included, and the model fit indices (*RMSEA* = 0.055 < 0.06, *se* = 0.002; *SRMR* = 0.077 < 0.08, *se* = 0.002) improved and implied an acceptable goodness-of-fit (Hu and Bentler 1998). Hence, considering the task-specific effects fit the data substantially better. One obvious difference between single measurement models and the full model occurred in the factor loadings of the non-targeted exploration indicators. In the full model, the latent non-targeted exploration could capture only the common features underlying Tasks 3 and 4, whose factor loadings exceeded 0.4.

Regarding the relationship between planning and problem-solving competency (i.e., *Q2a*), we begin by addressing the latent variable levels, namely their overall relationship. The correlation between latent planning and problem-solving competency was −0.093 (*p* = .007, *se* = 0.035). That is, the overall effect of planning on problem-solving was negative, but the magnitude of the effect was rather small. This result was similar to Eichmann et al.’s (2019) study, where the longest duration was not related to task success on average. For *Q2a* on the observed data level, namely the task-dependent relationships, Table 3 presents the relevant results that suggested the residual correlations were not negligible. Specifically, half of the residual correlations were positive, and the other half were negative. For Tasks 3, 4, and 5, after controlling for the latent variables in the model, spending more time on planning contributed to task performance, whereas spending more time on planning in Tasks 1, 6, and 7 impaired task performance. That is, the relationships between the longest duration indicator and task scores varied a lot across the tasks.

Regarding *Q2b*, as hypothesized, non-targeted exploration showed a strong positive relationship with problem-solving competency with a factor correlation equal to 0.887 (*p* < .001, *se* = 0.034). However, the answer to *Q2b* on the observed data level differed across tasks. The residual correlations between the responses and the non-targeted exploration indicators were significant and positive in the first three tasks but negative in Task 6 (see Table 3). That is, after considering the positive relationship between non-targeted exploration and problem-solving competency, different tasks showed distinct impacts on task performance. In addition, the residual correlations between the indicators of planning and non-targeted exploration by and large increased with the positions of the tasks. Engagement might be one explanation for this result. Specifically, participants who kept engaging in the assessment tended to invest more time in planning and more exploratory behaviors than those who gradually lost patience.

## 4. Discussion

In this article, we focused on planning, non-targeted exploration, and problem-solving competency using process measures and task performance in the 2012 PIAAC PS-TRE domain. We assessed the internal construct validity of the derived indicators and investigated their relationships using multidimensional latent variable analysis.

### 4.1. Summary of the Study

Our results provide additional evidence for the internal construct validity of the indicators of planning and problem-solving competency. It suggested that the latent planning greatly captured the common variance of the longest duration indicators and was relatively stable across tasks. However, the CFA results indicated that latent non-targeted exploration exerted varied influences on different tasks. The task interfaces can provide a potential explanation for the result. If the interfaces such as spreadsheets or emails contained features that are commonly used by respondents, it would likely be less necessary to explore these buttons to acquire new information. In contrast, novel information was embedded in a web environment in Tasks 3, 4, and 7, requiring potentially more non-targeted exploration, while Task 7 provided extra hints for non-necessary operations and thus prevented some non-targeted behaviors. In short, the familiarity of the presented environments and hints might weaken the influence of the latent non-targeted exploration.

After interpreting the internal construct validity of the process indicators, we then interpret the task-dependent relationships between planning and problem-solving competency. Task difficulty was not critical in explaining the diverse relationships after we inspected the task difficulty for each item provided by PIAAC (OECD 2013), a finding that was in line with Eichmann et al. (2019) who used the PISA 2012 problem-solving tasks. Instead, more specific task features can provide some insights. If some tasks (e.g., Task 4) require respondents to integrate complex information, investing more time in planning helps problem-solving (Mumford et al. 2001). Moreover, the relevance of information also mattered. Being stuck with irrelevant information can lead to biased planning (Mumford et al. 2001). For instance, we found that unsuccessful respondents tended to spend the longest duration on irrelevant emails compared with successful respondents in Task 6.

The other research interest of the study is the relationships between problem-solving competency and non-targeted exploration. The positive overall relationship between non-targeted exploration and problem-solving competency on the latent trait level indicated that non-targeted exploration facilitated representing and further contributed to successful task completion (Dormann and Frese 1994; Kapur 2008). However, the negative residual correlation for Task 6 implied that exploring too much was detrimental to solving the task. Paying too much attention to irrelevant information might complicate the problem and result in cognitive overload (Frese and Keith 2015). A common pattern for successful problem-solving involved actively trying some non-targeted operations or goal-directed behaviors to expand the problem space, distinguishing the features of these operations, and focusing on goal-directed behaviors to reach the desired state.

### 4.2. Contributions and Limitations

This article offers several contributions. From a theoretical perspective, we examined the internal construct validity of process indicators across multiple tasks, whereas many relevant studies have been limited to single items (e.g., Ulitzsch et al. 2021). Combining data from multiple tasks utilizes the information from the assessment to a greater extent and potentially provides more evidence for the stability of the conclusions. We found that the process indicators differed in the extent of internal construct validity, which suggested that researchers should carefully consider applying the measures from one task to another task even though both tasks are designed to measure the same concept. For practitioners, the longest duration can be employed as a good indicator for planning in other information-processing problems similar to the PS-TRE tasks, whereas non-targeted exploration would be less suitable to apply to routine problems with little novel information. On the contrary, if the task is rich in new information that respondents can explore to acquire, the amount of non-targeted exploration would be able to capture the common pattern of exploratory behaviors. 

Regarding the research topics, our results provide evidence for the functions of planning and non-targeted exploration in problem-solving based on human–computer interactions, deepening the understanding of their relationships in dynamic problems. The insight into the processes of complex problem-solving is crucial for educational systems since one important mission of education is to prepare students to become better problem-solvers (OECD 2014). Our results can potentially facilitate educational practice aiming at improving problem-solving skills. For example, it would be promising to implement a computer-simulated agent to help problem-solvers in terms of planning and non-targeted exploration. Specifically, if an individual has spent a long time planning in a dynamic problem without interacting with the task environment, the agent can offer a hint to encourage exploratory behaviors if the individual is not familiar with the task environment. In another circumstance, if an individual engaged in too much non-targeted exploration rapidly, the agent can advise spending more time on planning a strategy when the task requires respondents to incorporate complex information. Besides the development of digital tools, test developers can also compare the relationships between planning, non-targeted exploration, and task performance with the desired design to reflect on the task design. For example, if a task is designed to benefit from planning, the relationship between the longest duration and task performance should be positive; otherwise, test developers would need to reconsider their design. 

Some limitations of this study should also be noted. First, the indicator of non-targeted exploration requires researchers to define goal-directed and non-targeted operations that can be difficult for some types of problems. Second, the longest duration indicator reflects only the quantity of the planning, which does not necessarily imply the quality of the planning. Future studies can assess the quality of plans in dynamic problems and examine their relationship with task performance. In addition, similar to Eichmann et al. (2019), our definition of planning is broad in nature. Although we excluded the durations at the end of the tasks (e.g., reflecting process) in identifying the planning process, the longest duration can actually refer to the monitoring process. Third, although our indicators were based on previous studies, the underlying meaning of the latent variables must be interpreted carefully. Fourth, the current data are from the 2012 PIAAC PS-TRE domain, the core of which is information-processing skills (Greiff et al. 2017). However, other international assessments have various focuses, which may show different relationships between planning, non-targeted exploration, and problem-solving competency.

## 5. Conclusions

This study derived process indicators of planning and non-targeted exploration from the existing literature (Eichmann et al. 2019, 2020a, 2020b). Our results provide evidence for the internal construct validity of the planning indicator and response scores across multiple PS-TRE items, whereas the non-targeted exploration indicator was more challenging to be analyzed simultaneously across tasks when considering the dependency of the indicators from the same item. In addition, non-targeted exploration had a strong positive relationship with problem-solving competency. The results of residual correlations provided more detailed and diverse relationships between task performance, planning, and non-targeted exploration on the task level.

## Figures and Tables

**Table 1:** A simulated example of operation sequence and response times.

Operation	Notes	Time Interval	Planning Indicator	Exploration Indicator
START	Enter the problem system	-	-	System-defined
textlink_page1	Click on the first link	10 s	Yes	IniNT
toolbar_back_btn	Click on the back button in the toolbar	3 s	No	IniNT
web_menu_help	Click on the Help button in the menu	5 s	No	IniNT
textlink_page5	Click on the fifth link	8 s	No	GD
toolbar_bookmark_btn	Click on the bookmark button in the toolbar	7 s	No	GD
bookmark_add_page5	Confirm adding the fifth page to bookmark	4 s	No	GD
web_menu_help	Click on the Help button in the menu	3 s	No	RepNT
NEXT_INQUIRY	Request the next task	12 s	-	System-defined
END	End the task	4 s	-	System-defined

Note: IniNT = initial non-targeted. RepNT = repeated non-targeted. GD = goal-directed. We shortened the names of the operations in the raw log files.

**Table 2:** Standardized results for the single-factor models.

Variable	Estimate	SE	*p*
*Model 1a: Robust χ^2^ (35) = 56.179 (p = .013), RMSEA = 0.021 (se = 0.006), SRMR = .042 (se = 0.003)*			
P1	0.531	0.028	<.001
P2	0.648	0.025	<.001
P3	0.691	0.022	<.001
P4	0.662	0.025	<.001
P5	0.491	0.029	<.001
P6	0.639	0.027	<.001
P7	0.663	0.023	<.001
*Model 1b: Robust χ^2^ (42) = 52.208 (p = .134), RMSEA = 0.014 (se = 0.007), SRMR = .045 (se = 0.004)*			
E1	0.328	0.043	<.001
E2	0.264	0.045	<.001
E3	0.414	0.048	<.001
E4	0.611	0.056	<.001
E5	0.298	0.043	<.001
E6	0.179	0.046	<.001
E7	0.125	0.043	.003
*Model 1c: Robust χ^2^ (28) = 18.892 (p = .901), RMSEA < 0.001 (se = 0.003), SRMR = 0.020 (se = 0.003)*			
PS1	0.778	0.025	<.001
PS2	0.786	0.020	<.001
PS3	0.684	0.026	<.001
PS4	0.813	0.019	<.001
PS5	0.758	0.024	<.001
PS6	0.636	0.025	<.001
PS7	0.723	0.022	<.001

Note: P = the planning indicator; E = the non-targeted exploration indicator; PS = the problem-solving indicator.

**Table 3:** Standardized results of the residual correlations in Model 2.

Variable	Estimate	SE	*p*
PS1 with P1	−0.374	0.037	<.001
PS2 with P2	−0.068	0.034	.365
PS3 with P3	0.249	0.035	<.001
PS4 with P4	0.569	0.033	<.001
PS5 with P5	0.609	0.034	<.001
PS6 with P6	−0.181	0.035	.002
PS7 with P7	−0.155	0.033	.013
PS1 with E1	0.127	0.033	.014
PS2 with E2	0.234	0.032	<.001
PS3 with E3	0.179	0.024	<.001
PS4 with E4	0.066	0.030	.299
PS5 with E5	0.044	0.034	.428
PS6 with E6	−0.796	0.025	<.001
PS7 with E7	−0.038	0.032	.408
P1 with E1	−0.076	0.033	.057
P2 with E2	−0.002	0.033	.973
P3 with E3	0.059	0.028	.233
P4 with E4	0.240	0.031	<.001
P5 with E5	0.220	0.031	<.001
P6 with E6	0.120	0.034	.007
P7 with E7	0.208	0.032	<.001

Note: P = the planning indicator; E = the non-targeted exploration indicator; PS = the problem-solving indicator.

## Data Availability

The data used in this article can be found in the GESIS Data Catalogue (https://search.gesis.org/research_data/ZA6712?doi=10.4232/1.12955, accessed on 19 October 2021).

## Appendix A

The recoding rules for log-events:

The log-events were recoded using the following rules:

- We kept only the events implemented by the respondent and deleted the system events triggered by the respondent’s interaction event. For instance, when a respondent clicked on the “Add page” button in the bookmark pop-up window, three events were logged with the same timestamps: BOOKMARK_ADD, BUTTON, and DOACTION. In this case, we kept only BOOKMARK_ADD because it was sufficient for describing the operation implemented by the respondent.
- We aggregated the event type KEYPRESS. When a key is pressed, a KEYPRESS event with an ASCII value is logged. Because typing a string (e.g., a name) is regarded as a single operation, we aggregated consecutive KEYPRESS events as a single KEYPRESS event.
- All events from a combo-box (e.g., a SORT pop-up window) with several sorting rules were aggregated according to the final state of the SORT window.

## Appendix B

**Table A1:** Descriptive statistics for the raw process indicators without transformations.

Raw Indicator	Mean	SD	Min	Max	Skewness	Kurtosis
P1	66.75	59.85	1.09	1149	7.15	101.14
P2	55.63	56.10	4.46	1317	11.51	227.32
P3	41.04	29.33	1.91	432	4.67	42.70
P4	43.32	78.19	6.12	2421	24.79	739.48
P5	48.58	33.65	1.85	313	2.41	9.84
P6	34.99	322.84	4.25	10847	33.30	1112.34
P7	27.88	65.25	3.66	2157	28.70	921.94
E1	1.94	2.28	0	38	4.61	52.76
E2	8.50	18.90	0	204	5.82	44.57
E3	7.44	3.98	0	17	.07	−1.11
E4	6.48	5.15	0	35	1.08	1.49
E5	3.81	3.56	0	30	1.91	6.51
E6	8.37	10.68	0	65	1.27	.63
E7	3.6	5.56	0	46	3.21	13.26

Note: P = the planning indicator. E = the non-targeted exploration indicator.
